# Label-Free Proteomics Revealed Oxidative Stress and Inflammation as Factors That Enhance Chemoresistance in Luminal Breast Cancer

**DOI:** 10.1155/2019/5357649

**Published:** 2019-08-08

**Authors:** Bruno R. B. Pires, Carolina Panis, Vinícius Dias Alves, Ana C. S. A. Herrera, Renata Binato, Luciana Pizzatti, Rubens Cecchini, Eliana Abdelhay

**Affiliations:** ^1^Laboratório de Célula Tronco, Instituto Nacional de Câncer, Rio de Janeiro, RJ 20230-130, Brazil; ^2^Laboratório de Mediadores Inflamatórios, Universidade Estadual do Oeste do Paraná, Francisco Beltrão, PR 85605-010, Brazil; ^3^Instituto do Câncer de Londrina, Londrina, PR 86015-520, Brazil; ^4^Laboratório de Biologia Molecular e Proteômica do Sangue, Instituto de Química, Universidade Federal do Rio de Janeiro, Rio de Janeiro, RJ 21941-598, Brazil; ^5^Laboratório de Fisiopatologia e Radicais Livres, Universidade Estadual de Londrina, Londrina, PR 86057-970, Brazil

## Abstract

Breast cancer is the leading cause of cancer-associated death among women worldwide. Its high mortality rate is related to resistance towards chemotherapies, which is one of the major challenges of breast cancer research. In this study, we used label-free mass spectrometry- (MS-) based proteomics to investigate the differences between circulating proteins in the plasma of patients with chemoresponsive and chemoresistant luminal A breast cancer. MS analysis revealed 205 differentially expressed proteins. Furthermore, we used in silico tools to build protein-protein interaction networks. Most of the upregulated proteins in the chemoresistant group were closely related and tightly linked. The predominant networks were related to oxidative stress, the inflammatory response, and the complement cascade. Through this analysis, we identified inflammation and oxidative stress as central processes of breast cancer chemoresistance. Furthermore, we confirmed our hypothesis by evaluating oxidative stress and performing cytokine profiling in our cohort. The connections among oxidative stress, inflammation, and the complement system described in our study seem to indicate a pivotal axis in breast cancer chemoresistance. Hence, these findings will have significant clinical implications for improving therapies to bypass breast cancer chemoresistance in the future.

## 1. Introduction

Breast cancer (BC) is the leading cause of cancer-associated death among women worldwide. In the U.S., approximately 270,000 new cases of female BC and more than 40,000 deaths are expected in 2019 [1]. More than 70% of all diagnosed BC cases are estrogen- and/or progesterone receptor-positive (ER+ and PR+, respectively), which is defined as the luminal subtype [2]. Over the past two decades, the investigation of BC biology has increased our understanding of BC at the molecular level. However, relevant issues remain to be addressed. In this context, resistance to treatment is considered the main critical challenge in BC research since resistance is responsible for treatment failure, especially in cases of metastatic disease [3].

Cytotoxic chemotherapy for BC treatment is based on a protocol that includes taxanes and anthracyclines, such as the combined paclitaxel/doxorubicin treatment. Paclitaxel belongs to the taxane family and acts by stabilizing microtubules, altering cell division and, consequently, causing cell death [4]. An additional mechanism of this drug is the generation of oxidative stress and inflammatory mediators [5]. Studies have shown that paclitaxel promotes cytotoxicity by reactive oxygen and nitrogen species (ROS and RNS, respectively) [6]. Doxorubicin is the most commonly used anthracycline in BC treatment. It disrupts DNA replication by binding to topoisomerase II and generating free radicals, resulting in DNA damage [7]. In both cases, oxidative stress is generated when there is an imbalance between the production of antioxidant substances by cancer cells and the production of ROS by chemotherapeutics. Approximately 50% of patients treated with cytotoxic chemotherapy develop resistance to treatment within a 6-month interval [8–11]. Chemoresistance is defined as tumor resistance intrinsic or extrinsic to the chemotherapeutic treatment leading to recurrence of the disease or its progression to metastasis [12]. Thus, chemoresistance poses one of the major challenges in BC research [13].

The early detection of chemoresistance has a significant effect on reducing mortality. Proteomics is a powerful high-throughput tool for screening circulating proteins and evaluating the response to treatment or disease recurrence [14, 15]; however, chemoresistance in BC has not been sufficiently explored. Many chemoresistance studies are cell line-based, which does not replicate the complexity of the human body. Hence, discovering proteomic signatures associated with chemoresistance is critical to differentiate chemoresistant and treatment-sensitive patients.

Our group recently suggested markers of BC progression through plasma proteomic profile analyses [16]. In the present study, resistance to combined paclitaxel/doxorubicin treatment in luminal A breast cancer patients was investigated using a label-free proteomic approach to acquire a comprehensive analysis of the crucial factors related to this phenomenon. Our findings revealed that most of the upregulated proteins in the chemoresistant group are closely related and tightly linked. Although our results showed a strong interplay between inflammation and oxidative stress in the chemoresistant condition, the complement system might be responsible for their connection, which has been well demonstrated in age-related macular degeneration [17], but not for breast cancer yet. Through this analysis, we identified inflammation and oxidative stress as central signaling pathways and possible markers associated with BC chemoresistance. In addition, to the best of our knowledge, this is the first in-depth proteomic study of the differentially circulating proteins in patients with BC chemoresistance. These findings will have critical implications for the development of more effective therapies for BC.

## 2. Materials and Methods

### 2.1. Design of the Study and Patient Characteristics

**Figure 1:**
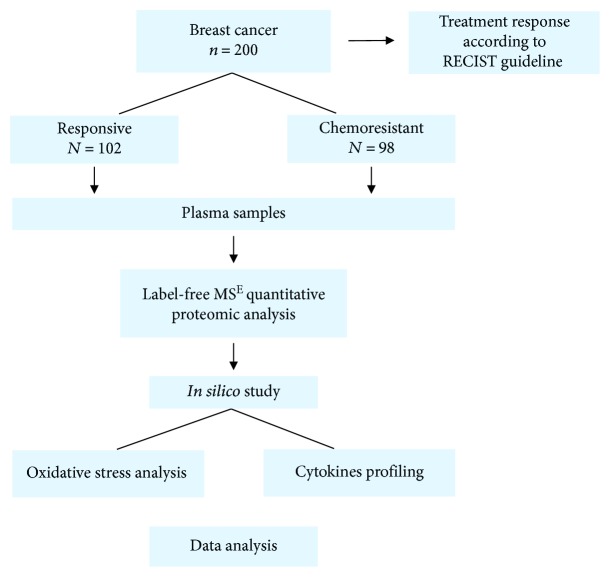
Schematic design of the study.

Two hundred women diagnosed with invasive breast carcinoma who attended a public Oncology Center in Brazil were enrolled in this study. This was a prospective study that started in 2014 with follow-up until 2018.  Figure 1 displays a schematic design of this study, which was approved by the Research Ethics Committee of the Institution and the National Ethics Research Council (CAAE 23753014.3.0000.5231). All participants signed informed consent forms.

Patients were included in the study from the time of diagnosis. They were administered 5-6 cycles of combined paclitaxel (175 mg/m^2^) and doxorubicin (60 mg/m^2^) every 21 weeks. Samples were collected at diagnosis before starting treatment and posteriorly categorized according to the pattern of each patient's chemotherapy response in the first year of treatment. Thereafter, patients were categorized into the following groups according to the treatment response criteria established by the Response Evaluation Criteria in Solid Tumors (RECIST) guidelines [18]: (1) patients responsive to chemotherapy and (2) patients resistant to chemotherapy. All patients were subjected to the same treatment schedule, which included anthracyclines and taxanes, and were evaluated at the end of the first-choice treatment module (5-6 months). The following parameters in the cohort were considered for clinicopathological characterization: age at diagnosis, weight, height, comorbidities, International Union Against Cancer (UICC) tumor, node, metastasis (TNM) stage, hormonal status of the tumors, and chemotherapy protocol. Patients bearing tumors exhibiting amplification of the receptor of epidermal growth factor 2 (HER2) were excluded from this study due to the use of other treatment protocols (monoclonal anti-HER2 antibodies). Other exclusion criteria were a history of previous chemotherapy, smoking, hepatic, renal or cardiac dysfunction, diabetes, and other chronic conditions that could interfere in the analysis of the results. Clinicopathological data were obtained from medical records and are presented in Table 1.

### 2.2. Plasma Collection and In-Solution Tryptic Digestion

Whole blood samples (20 mL) were obtained by peripheral venipuncture and collected in sodium EDTA tubes. The tubes were centrifuged for 5 min at 1400 × g at 4°C, and the nondepleted plasma was then collected, supplemented with a 1 : 1000 (*μ*L) protease inhibitor cocktail (GE Healthcare, USA), and stored at −80°C. Nondepleted plasma samples were used to prevent loss of information during the removal of the high-abundance proteins. Protein concentration was determined using the Bradford assay. Proteomic analysis was performed using pooled plasma samples (500 *μ*L from each individual sample) for each group (responsive and chemoresistant patients), and 1 mg of nondepleted plasma samples were concentrated 39-fold and exchanged into 50 mM ammonium bicarbonate (NH_4_HCO_3_) using a 3 kDa ultrafiltration device (Millipore, USA). Then, 200 *μ*g of protein was denatured (0.1% RapiGEST SF at 60°C for 15 min) (Waters, USA), reduced with 10 mM DTT (60°C for 30 min), alkylated with 10 mM iodoacetamide (30 min at room temperature in the dark), and, after that, enzymatically digested with trypsin at a 1 : 50 *w*/*w* enzyme/protein ratio (Promega, USA), according to the method described by Panis et al. [19]. Digestion was stopped by adding 10 *μ*L of 5% TFA, and yeast alcohol dehydrogenase (ADH; P00330, Waters) was added to the digests at a final concentration of 10 fmol/*μ*L as an internal standard for absolute quantification [20].

### 2.3. Label-Free Protein Quantitation via Mass Spectrometry

For qualitative and quantitative experiments, the nanoUPLC tandem nanoESI-HDMS^E^ proteomic approach was applied in this study. A nanoACQUITY UPLC system (Waters, UK) was used according to the method described by Panis et al. [16].

For the first dimension, a strong cation exchange (SCX) column was used. The samples were eluted from the SCX column using nine salt gradient fractions that were followed by a reversed-phase (RP) gradient. The released peptides were captured by a downstream RP trap column. After all the peptides had been captured, the trap column was placed online with a different RP analytical column, and an RP gradient of 5–40% acetonitrile (containing 0.1% *v*/*v* formic acid) over 58 min with a flow rate of 600 nL/min was used as the second dimension. Analyses were performed using nanoelectrospray ionization in positive ion mode nanoESI (+) and a NanoLockSpray ionization source (Waters, UK). Multiplexed data-independent (DIA) scanning with specificity and selectivity based on nonlinear “T-wave” ion mobility (HDMS^E^) experiments was performed with a Synapt HDMS mass spectrometer (Waters, UK) as previously described [16]. Full-scan orthogonal acceleration time-of-flight (oa-TOF) MSE was acquired from an *m*/*z* of 50 to 2000.

### 2.4. Database Searching, Protein Quantification, and In Silico Analysis

Database searching and protein quantification were performed as previously reported [16] using ProteinLynx Global Server v.2.5.2 (PLGS) and Expression^E^ informatics. Proteins present in all replicates of each condition were considered for expression analysis using the Expression^E^ tool. The identified proteins were organized into a statistically significant list corresponding to increased and decreased regulation ratios between samples from patients with the chemoresistant group vs. the chemosensitive group. Additional filtering procedure was performed to select only those proteins that presented differential expression levels (ratios) with *p* value less than 0.05. Next, in silico analysis was performed using STRING v.10 software (http://string-db.org) [21], the PANTHER (http://pantherdb.org) [22], KEGG (http://genome.jp/kegg) [23], and IPA (QIAGEN Inc., https://www.qiagenbioinformatics.com/products/ingenuity-pathway-analysis) [24] to identify the main interaction networks, biological processes, and signaling pathways corresponding to the differentially expressed proteins.

### 2.5. Oxidative Stress Analyses

To evaluate oxidative stress in the plasma, we determined the carbonyl content, malondialdehyde (MDA), and nitrite levels as estimates of nitric oxide (NO) and the antioxidant profile by measuring the total reactive antioxidant potential (TRAP) and reduced glutathione (GSH) levels. Healthy control plasma samples (*n* = 32) were included as reference.

The carbonyl content was measured as an estimate of oxidative injury to proteins, as previously described [25]. Dinitrophenylhydrazine (DNPH 10 Mm in HCl 2.5 M) was added to 200 *μ*L of plasma, which was incubated in an ice bath (1 hour) and successively incubated with trichloroacetic acid 20% on ice for 15 minutes. Next, the samples were centrifuged (3000 rpm, 15 min), the supernatants were discarded, and the pellets were treated twice with an ethanol/water (1 : 1) solution. The final precipitates were dissolved in guanidine 6 M pH 2.3 and incubated for 24 h at 37°C [26]. The carbonyl content was calculated by obtaining spectra from 355 to 390 nm of the DNPH-treated samples. The obtained peaks were employed to calculate the carbonyl concentration using a molar extinction coefficient of 22 M^−1^ cm^−1^. The results are expressed in nmol/mL/mg total protein. To determine the carbonyl content, total protein levels were measured with Folin-Ciocalteu reagent [27].

Malondialdehyde (MDA) levels were determined by high-performance liquid chromatography (HPLC) by using an HPLC-20AT Shimadzu equipped with an LC20AT pump and SPDM20A UV diode array absorbance detector employing a C18 reversed-phase column, as previously described [28]. Aliquots of 160 *μ*L of plasma samples or standard solution reacted with 100 *μ*L of 0.5 M perchloric acid. Samples were centrifuged for 5 min at 5000 × g at 4°C. 180 *μ*L of supernatant was recovered to react with 100 *μ*L of thiobarbituric acid for 30 min at 95°C. Reaction was stopped by ice bath, and 100 *μ*L of 1 M NaH_2_PO_4_, pH 7.0, was added to stabilize sample pH. Further, samples were centrifuged for 10 min at 5000 × g at 4°C. The mobile phase consisted of 65% 50 mM KH_2_PO_4_ buffer, pH 7.0, and 35% HPLC-grade methanol. To determine MDA concentration, a standard curve was performed. For preparation of standard solution of MDA, 10 mL of 0.1 M HCl was added in 10 mL of 1,1,3,3-tetraethoxypropane (TEP), and this solution was maintained for 5 min in boiling water, following ice bath to complete synthesis of MDA. Readings were taken at 535 nm for 12 min with an isocratic flow of 0.8 mL/minute, and the results are expressed as nM MDA.

Nitrite levels were determined as estimates of the NO content and determined as previously described by Herrera and colleagues [29]. Plasma aliquots of 60 *μ*L were deproteinized by adding 50 *μ*L of 75 mM ZnSO_4_ solution and after centrifugation (9500 × g for 2 min at 25°C) were mixed with 55 mM NaOH. The supernatant was recovered and diluted in a glycine buffer 5 : 1 in 45 g/L glycine, pH 9.7, with further incubation with cadmium granules activated in 5 mM CuSO_4_ in 15 g/L glycine-NaOH buffer, pH 9.7 by 5 min. Aliquots of the recovered supernatant were mixed with the same volume of Griess reagent. A calibration curve was prepared by dilution of NaNO_2_ in distilled sterile water. The absorbance was measured at 550 nm on a standard microplate reader, and the results are expressed as *μ*M nitrite.

For antioxidant profiling, the total reactive antioxidant potential (TRAP) was determined, as described by Repetto and colleagues [30]; 2,2′-azobis (ABAP) was employed as a radical generator, and luminol was used to amplify photon detection and light emission by chemiluminescence. ABAP basal emission (900 *μ*L of glycine buffer 0.1 M pH 8.6, 50 *μ*L of luminol and 50 *μ*L of ABAP) and hydrosoluble vitamin E standard solution (trolox, 6-hydroxy-2,5,7,8-tetramethylchroman-2-carboxylic acid 25 *μ*M, 830 *μ*L of glycine buffer 0.1 M pH 8.6, 70 *μ*L of trolox, 50 *μ*L of luminol, and 50 *μ*L ABAP) emissions were recorded as references. For sample analysis, plasma was diluted 1 : 50 (830 *μ*L of glycine buffer 0.1 M pH 8.6, 70 *μ*L of sample, 50 *μ*L of luminol, and 50 *μ*L of ABAP). All readings were performed in a GloMax luminometer (Promega, USA) during 30 minutes, 5 readings/second. Results were expressed as nM sample equivalents of trolox.

GSH content was determined as described by Sedlak and Lindsay [31]. Plasma aliquots (60 *μ*L) were deproteinized with 250 *μ*L of trichloroacetic acid 50% and centrifuged at 2400 × g for 15 min, and the supernatants were added to 2 mL of 0.4 M TRIS buffer, pH 8.9. This mixture reacted with 50 *μ*L of 5,5′-dithiobis (2-nitrobenzoic acid) solution. A standard curve was performed in order to determine GSH concentration in samples. The absorbance was read at 412 nm, and results were expressed in nM.

### 2.6. Cytokine Analysis

Interleukin-12 (IL-12), interleukin-10 (IL-10), transforming growth factor beta (TGF-*β*1), and tumor necrosis factor alpha (TNF-*α*) levels in plasma samples were determined by using a commercial antibody-specific RSG ELISA kit (eBioscience, USA). The results were calculated in pg/mL by fitting to a standard curve obtained using recombinant human cytokines. Healthy control plasma samples (*n* = 32) were included as reference.

### 2.7. Statistical Analysis

Analyses were conducted in duplicate, and the data are expressed as the means ± error of the means. Oxidative stress and cytokine parameters were compared by unpaired Student's *t*-test (parametric data) or the Mann-Whitney test (nonparametric data). A *p* value < 0.05 indicated significance. All statistical analyses were performed using GraphPad Prism 7.0 software (GraphPad Software, San Diego, CA).

## 3. Results

### 3.1. Clinicopathological Data

Table 1 shows the clinicopathological data of the patients. The mean age at diagnosis was 56.3 years, and most of the patients presented advanced disease, poorly differentiated tumors, and hormone-positive breast tumors larger than 2 cm.

### 3.2. Proteomic Profile of Breast Cancer Chemoresistance

To identify the differentially expressed proteins in plasma samples from patients with chemoresistant BC vs. patients with chemosensitive BC treated with a combination of doxorubicin and paclitaxel, we used label-free protein quantitation by MS. Proteomic screening revealed 444 proteins in the plasma samples from chemoresistant patients and 482 proteins in plasma samples from chemosensitive patients, of which 205 were differentially expressed between the two conditions. The total number of identified protein was separated into unique (exclusive in each condition) and differentially expressed (Table 2).

To identify the main biological processes and signaling pathways associated with the differentially expressed proteins, we performed separate in silico analyses of the upregulated and downregulated proteins. The most relevant processes associated with the differentially expressed proteins were oxidative metabolism, immune response (including inflammation, the humoral response, and the complement system), blood coagulation, cytoskeleton remodeling/cell adhesion, and DNA repair/kinetochore assembly. The proteins associated with these processes are shown in Table 3 (upregulated) and Table 4 (downregulated).

We found increased levels of proteins relevant to migratory behavior, such as Rho GTPase-activating protein, fibronectin, and vitronectin, in the chemoresistant samples compared with their levels in the responsive samples. Changes in the levels of cytoskeleton proteins and proteins that interact with the extracellular matrix (ECM) play an essential role in the invasive phenotype and progression of cancer. Consistently, we observed decreased levels of the adhesion proteins collagen alpha-1(VII), integrin alpha V, and keratin type II. The levels of proteins associated with DNA repair and kinetochore assembly were also changed. We observed the increased expression of DnaJ homolog subfamily C member 10, LINE-1 type transposase domain-containing protein 1, and centromere/kinetochore protein zw10 homolog, although we identified the decreased expression of DNA polymerase alpha catalytic subunit and centromere protein F, among others. Blood coagulation was represented through upregulation of plasminogen, prothrombin, alpha 1-antitrypsin, antithrombin III, and kininogen 1, among others, and downregulation of fibrinogen *α* and fibrinogen *β*. We identified several oxidative metabolism-associated proteins with altered expression in the resistant samples, indicating that oxidative metabolism may be a critical biological process for chemoresistance. The levels of iron metabolism-related proteins haptoglobin, hemoglobin subunits *α* and *β*, hemopexin, serotransferrin, and ceruloplasmin were increased. We also observed augmented levels of proteins related to the modulation of oxidative stress and vitamins, such as afamin and vitamin D-binding protein. Another biological process that was shown to be relevant was the immune response. Several immunoglobulins were upregulated in the resistant samples compared with their expression in the responsive samples. The same was observed for complement cascade proteins, including cascade initiators (C1q subunits, C4, C3, and complement factor B) and effectors (C5). Inflammatory and acute phase proteins were also differentially expressed in the chemoresistant samples. We observed upregulation of lumican, C-reactive protein, and apolipoproteins, whereas AKT3 and RGS14 were downregulated.

**Figure 2:**
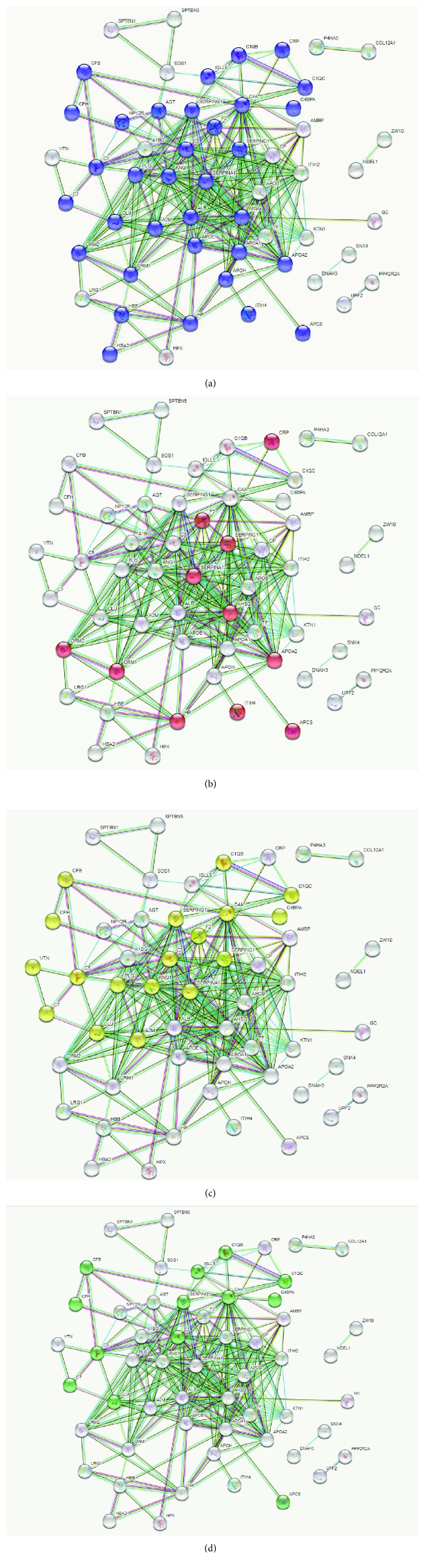
Network of interactions among the upregulated proteins in chemoresistant breast cancer identified by STRING software. (a) Proteins identified in the representative “response to oxidative stress” network are in blue. (b) Proteins identified in the representative “acute inflammatory response” network are in red. (c) Proteins identified in the representative “complement and coagulation cascades” network are in yellow. (d) Proteins identified in the representative “innate immune system” network are in green. The networks were generated with high interaction score > 0.9.

**Figure 3:**
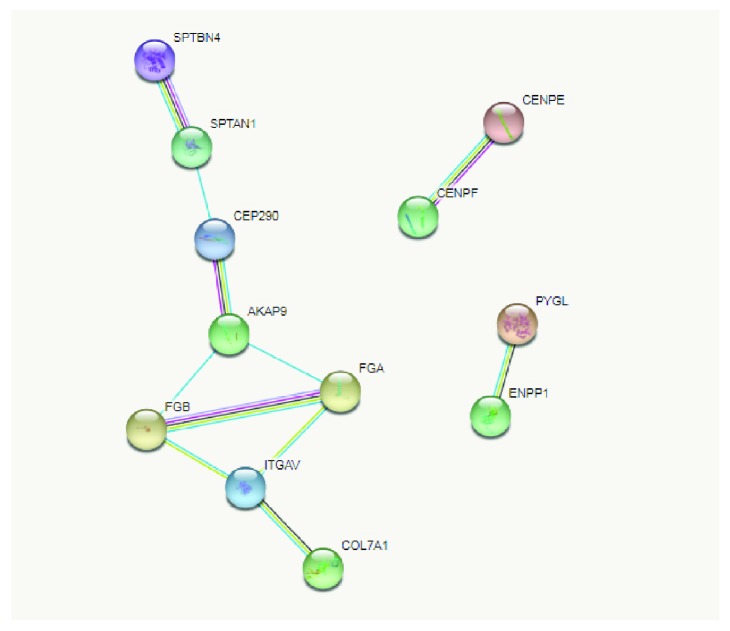
Network of interactions among the downregulated proteins in chemoresistant breast cancer identified by STRING software. Proteins were clustered according to the main representative networks identified. The networks were generated with high interaction score > 0.9.

Based on the most relevant biological processes and pathways revealed in our analysis, STRING software was used to build networks for the lists of up- and downregulated proteins (Figures 2 and 3, respectively). We observed that most of the upregulated proteins were associated with more than one biological process. This provoked network connection among the processes in STRING analysis. The majority of the upregulated proteins could be classified into the 4 following biological networks directly associated with processes relevant to BC chemoresistance: “response to oxidative stress” (Figure 2(a)), “acute inflammatory response” (Figure 2(b)), “complement and coagulation cascades” (Figure 2(c)), and “innate immune system” (Figure 2(d)). In contrast, the same analysis of the downregulated proteins showed distinct roles for each member and clustered them into exclusive networks (Figure 3). Nevertheless, they revealed a direct connection by their association with different biological processes, such as the cytoskeleton organization-fibrinolysis axis. To obtain a more accurate view of the molecular changes in samples of chemoresistant patients, we used the IPA software to identify the networks and canonical pathways most altered in this condition. The data are shown in the Supplementary Figures S1-6.

### 3.3. Oxidative Stress and Inflammatory Profile of Chemoresistant Breast Cancer

Since the proteins identified by proteomic analysis revealed the significance of inflammation and oxidative stress, we sought to investigate whether such processes were altered in the chemoresistant group.

**Figure 4:**
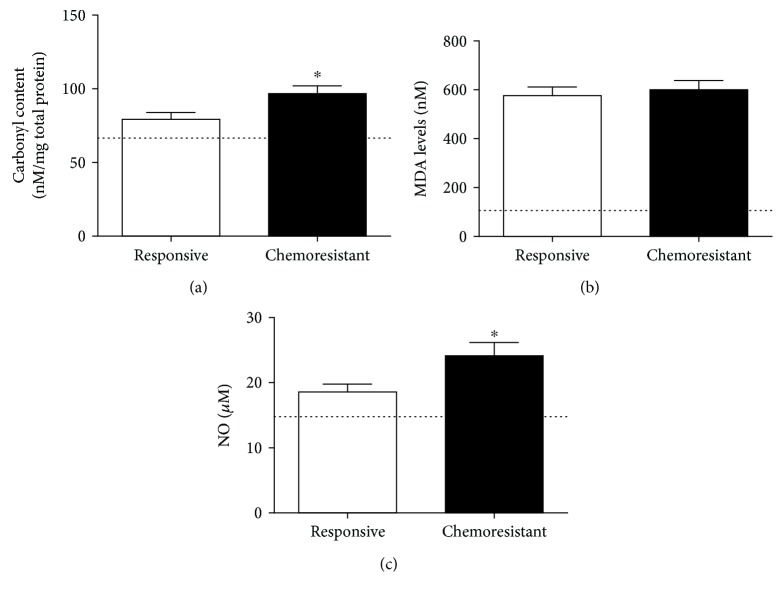
Prooxidant parameters in plasma samples from responsive and chemoresistant patients. Carbonyl content (a), malondialdehyde levels (MDA, (b)) and nitric oxide content (NO, (c)) were measured to determine the prooxidant profile of both groups. ^∗^ indicates a significant difference (*p* < 0.05). The line illustrates the mean levels of each parameter as determined in healthy controls.

Figure 4 shows the prooxidant parameters. The carbonyl content (Figure 4(a)) was higher in the chemoresistant patients than in the responsive patients (79.24 ± 4.68 nM/mg total protein in the responsive group and 96.72 ± 5.27 nM/mg total protein in the chemoresistant group, *p* = 0.0160). No variations were found in the MDA levels (576.3 ± 38.15 nM in the responsive group and 600.4 ± 38.2 nM in the chemoresistant group, *p* = 0.6456, Figure 4(b)). NO levels (Figure 4(c)) were also augmented in the chemoresistant group compared with the responsive group (18.59 ± 1.19 *μ*M in the responsive group and 24.15 ± 2.0 *μ*M in the chemoresistant group, *p* = 0.0486).

**Figure 5:**
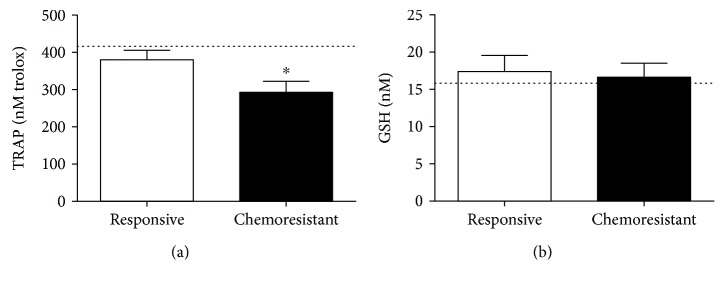
Antioxidant profiling of plasma samples from responsive and chemoresistant patients. The total radical antioxidant parameter (TRAP, (a)) and reduced glutathione (GSH, (b)) levels were measured to determine the antioxidant profile of both groups. ^∗^ indicates a significant difference (*p* < 0.05). The line illustrates the mean levels of each parameter as determined in healthy controls.

According to antioxidant profiling of the groups (Figure 5), the chemoresistant patients exhibited lower levels of TRAP (292.8 ± 29.6 nM trolox) than the responsive patients (380 ± 26.6 nM trolox, *p* = 0.0314, Figure 5(a)). No differences were detected in GSH levels between the two groups (17.4 ± 2.16 *μ*M in the responsive group and 16.7 ± 1.9 *μ*M in the chemoresistant group, *p* = 0.7918, Figure 5(b)).

**Figure 6:**
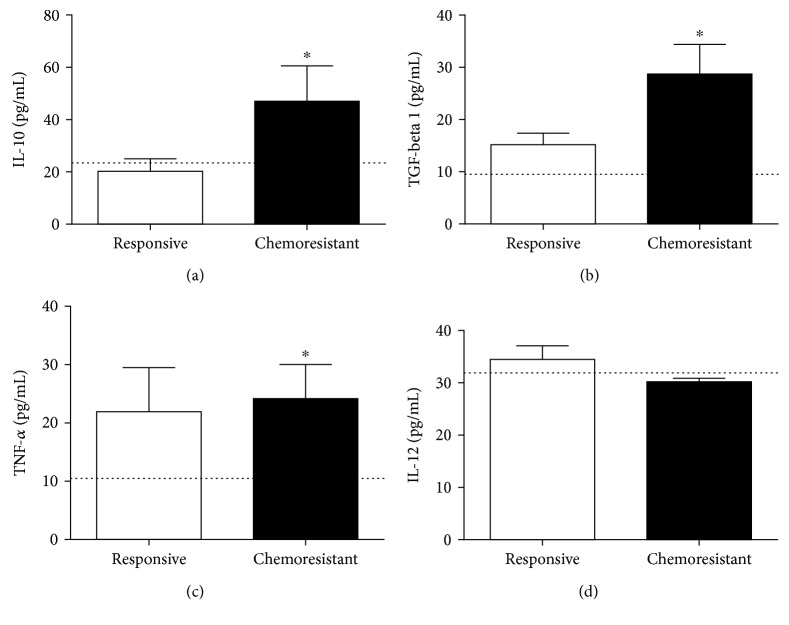
Cytokine profiling. The circulating levels of IL-10 (a), TGF-*β*1 (b), TNF-*α* (c), and IL-12 (d) were evaluated in both the responsive and resistant groups. ^∗^ indicates statistical significance (*p* < 0.05). The line illustrates the mean levels of each parameter as determined in healthy controls.

Cytokine measurement (Figure 6) revealed that the chemoresistant patients presented higher levels of IL-10 (20.23 ± 4.8 pg/mL and 47.06 ± 13.5 pg/mL, respectively, *p* = 0.0439, Figure 6(a)), TGF-*β*1 (15.18 ± 2.2 pg/mL and 28.71 ± 5.7 pg/mL, respectively, *p* = 0.025, Figure 6(b)), and TNF-*α* (21.9 ± 7.6 pg/mL and 25 ± 5.8 pg/mL, respectively, *p* = 0.0414, Figure 6(c)) than the responsive patients. No differences were observed in the IL-12 levels between the two groups (34.5 ± 2.6 pg/mL in the responsive group and 30.23 ± 0.67 pg/mL in the chemoresistant group, *p* = 0.41, Figure 6(d)).

For reference, we included healthy control levels for each parameter, represented as a line in the graphs, and the means were 67.2 nM/mg total proteins for carbonyl content (Figure 4(a)), 106 nM for MDA (Figure 4(b)), 14.6 *μ*M for NO (Figure 4(c)), 416 nM trolox for TRAP (Figure 5(a)), 15.6 nM for GSH (Figure 5(b)), 23 pg/mL for IL-10 (Figure 6(a)), 9.1 pg/mL for TGF-*β*1 (Figure 6(b)), 10.6 pg/mL for TNF-*α* (Figure 6(c)), and 31.9 pg/mL for IL-12 (Figure 6(d)).

In spite of one of the aims of the present study was to understand comparatively the differential redox profile between responsive and resistant patients, it can be noted that both groups exhibited different levels for all oxidative stress parameters when compared to the baseline of healthy controls. Moreover, chemoresistant patients presented important differences if compared to either responsive or healthy women.

## 4. Discussion

The main aspects associated with the high mortality rates of breast cancer are related to advanced stages of disease at diagnosis, the limited efficacy of treatment and resistance towards chemotherapy. Chemoresistance poses as one of the major challenges in breast cancer treatment [13], and its underlying molecular mechanisms remain unclear.

In the present study, we investigated the chemoresistance mechanisms in women with breast cancer carrying luminal A breast cancer by using the label-free proteomic approach. This strategy allows mapping the differential changes when comparing groups with distinct responses and indicates putative targets to further investigate and validate.

The main chemotherapy schedule used to treat the patients enrolled in this study was the combined paclitaxel/doxorubicin protocol, largely employed as the first line of treatment for luminal breast cancer worldwide. Beyond its main mechanism of action on cell microtubules [4], paclitaxel is known by generating oxidative stress and promoting changes in inflammatory mediator patterns [5, 6]. In the same way, doxorubicin acts as a DNA replication disruptor and gives rise to free radicals that results in DNA damage and cell death [7]. Despite these mechanism of actions, some tumors possess adaptative mechanisms that allow cell surviving and chemoresistance development.

A large number of chemoresistance studies are based on cell lines, which does not replicate the complexity of the human body. Thus, to the best of our knowledge, this is the first in-depth proteomic study that exploits the differential profiling of circulating proteins in breast cancer patients that undergo chemoresistance. It is important to highlight that all included patients had their samples collected at diagnosis, prior to any therapeutic intervention, and were categorized as responsive or resistant of the chemotherapeutic treatment. The data presented here indicate that it is possible to distinguish the systemic profile of patients still at diagnosis, when clinicians do not know if the patient will respond or not to the treatment. The initial goal of the present study was to determine the differences between the circulating proteomic profiles of chemoresistant and chemosensitive breast cancer patients. Furthermore, the identification of relevant biological processes and signaling networks shows that most of the upregulated proteins clustered into network connections, related to inflammation, redox signaling, and immune responses. Thus, we decided to further validate such pathways by measuring some proteins and metabolites resulting from the inflammatory axis and investigate if such targets correlated with the chemoresistant phenotype in breast cancer patients diagnosed with luminal A breast cancer.

The luminal A phenotype is known as the tumor with the best prognosis in breast cancer. In spite of this, some patients may progress as nonresponsive to treatment, and the reasons why this phenomenon occurs are not clear yet. A recent study from Zhang and colleagues reported some similarities with proteins found in our study. The authors compared plasma samples from ovarian cancer patients who were chemosensitive or chemoresistant by using a proteomic approach [32]. In accordance with our findings, the study found the upregulation of complement C4A, clusterin, and alpha 1-antitrypsin in the chemoresistant patients. These data suggest that some circulating proteins may be common players of chemoresistance, independent on the type of cancer.

In relation to the key processes identified in the present study, a body of evidence has shown the contribution of oxidative stress-related events to the physiopathology of breast cancer, including changes according to disease staging, types of treatment, and disease subtypes [11, 16, 19]. In the context of chemoresistance, the levels of antioxidants such as glutathione (GSH) play an essential role in the induction of chemoresistance. Reduced levels of GSH have been reported to enhance cellular sensitivity to anticancer-induced apoptosis. In contrast, elevated levels of antioxidant agents may confer resistance to drug-induced ROS [33]. Some abundant proteins identified here in the chemoresistant group, as albumin, ceruloplasmin, hemopexin, haptoglobins, and serotransferrin, play an important antioxidant role in plasma by sequestering iron ions [34]. Iron is a potent generator of oxidative stress, since it is a catalyst of Fenton's reaction that generates significant amounts of free radicals.

Several ROS and RNS can modulate signaling pathways that enhance the proinflammatory profile. Inflammatory cells liberate reactive species at the site of inflammation, as well as induce systemic changes in immune responses that lead to excessive oxidative stress [35]. Dysregulated inflammation is commonly associated with tissue damage, since in the inflammatory milieu, activated cells release proteases, reactive species, and chemical mediators (cytokines, chemokines, and complement components) [34]. As inflammation and oxidative stress can induce each other, a continuous, vicious cycle is commonly observed.

In the present study, oxidative stress analysis showed that the chemoresistant patients presented higher levels of carbonyls in association with augmented NO as well as impaired antioxidants. This scenario clearly indicates that these patients are more oxidatively/nitrosatively stressed than the responsive patients.

In recent years, redox signaling has been identified as a pivotal phenomenon in chemoresistance. In breast cancer, overexpression of the master regulator of redox homeostasis, NF-E2-related transcription factor 2 (Nrf2), in tumor cells was clearly implicated as a central mechanism of acquired chemoresistance [36]. The ability of Nrf2 to regulate chemotherapy sensitivity in BC is reflective of the antioxidant response element- (ARE-) bearing gene products regulated by this transcription factor, which function in cytoprotective responses [37]. Thus, antioxidant defense is the result of the balance between ARE-encoded enzymes and nonenzymatic antioxidants. Our results show that chemoresistant patients presented reduced levels of total nonenzymatic antioxidants in their plasma compared with their levels in chemosensitive patients. This fact may reflect both augmented systemic consumption and the demands of the tumor.

Carbonylation is a marker of the systemic oxidation of proteins [38] and plays a role in cell signaling [39]. Antioxidant consumption in the presence of protein carbonylation is expected in resistant cancer cells, and nitrosative stress participates in the generation of such products [40]. In the presence of inflammation, NO can react with superoxide anions from the mitochondria, yielding the most powerful reactive species, peroxynitrite [41]. The augmentation of NO in chemoresistant patients compared with chemosensitive patients suggests the activation of nitrosative stress in chemoresistant patients.

NO is a pleiotropic molecule with multiple functions and a dual role in redox and immune responses. Although NO is a classical tumoricidal molecule, altered NO homeostasis is related to chemoresistance [42, 43], and this mechanism seems to involve the protective effects of tumor-associated macrophages (TAMs) against proapoptotic events [44].

The cytokine panel from chemoresistant patients revealed here represents the sum of systemic cytokine balance. Tumors, and even immune cells, are constantly stimulated to produce and secrete such cytokines in cancer, albeit in a disordered manner. Our data show that chemoresistant patients simultaneously exhibit significantly higher levels of IL-10, TGF-*β*1, and TNF-*α* than chemosensitive patients, which contradicts the classical concept of an equilibrium between Th1/Th2 cytokines.

During immune responses, TNF-*α* is initially produced to fight cancer cells. However, when immune cells infiltrate the tumor mass, this activity inverts, and the production of TNF-*α* benefits to the tumor progression [45]. This controversial behavior of TNF-*α* also suggests a role for this molecule in the acquisition of chemoresistance. ER-positive breast cancer cells that resist TNF-*α*-induced death are associated with a multidrug-resistant phenotype by epithelial-mesenchymal transition- (EMT-) driven mechanisms [46]. Furthermore, circulating TGF-*β*1 was also increased in chemoresistant patients compared with chemosensitive patients. In breast cancer, TGF-*β*1 antagonizes ER-*α* signaling by inducing EMT and chemoresistance [47]. Similarly, IL-10 produced by TAMs can induce breast cancer chemoresistance [48]. Collectively, these findings support the hypothesis that the sustained circulation of TNF-*α*, TGF-*β*1, and Il-10 observed here constitute a putative synergistic mechanism of chemoresistance induction and maintenance, in addition to strongly supporting the perpetuation of oxidative stress [11, 49].

Although inflammation and oxidative stress dominate the chemoresistant signature presented in our study, the complement system might be responsible for their connection. In recent years, the paradigm regarding the role of complement proteins in the context of cancer has been broken. Some studies found that these proteins may be associated with ovarian and BC progression. However, the mechanism of this has not yet been described [50, 51].

Dysregulation of the complement system leads to autologous damage, and the complement system has been implicated in the pathogenesis of a wide spectrum of diseases [50, 51]. We observed the upregulation of several complement proteins in chemoresistant samples compared with chemosensitive samples, including members of the classical (C1q, C4, and C4b) and alternative (C3, factor H) pathways and the C5 effector.

Complement C3 is a key complement protein. The deposition of C3 and C3b on the endothelium increased oxidative stress in retinal vessels [17]. Bonavita and colleagues reported that C3-deficient mice were protected against carcinogen-induced cancer because of reduced inflammation [52]. C5 also plays a central role in the complement cascade. Beyond forming the terminal complement complex, called the membrane-attack complex (MAC, C5b-9), C5 has been reported to play an essential proinflammatory role [53]. Conversely, C1 inhibitor (SERPING1) was upregulated in chemoresistant samples compared with chemosensitive samples, showing a balance between induction and repression of the complement cascade. This inhibitor forms stable complexes with C1 subunits, which results in the repression of classical complement pathways. In addition, SERPING1 inhibits the inflammation, clotting, and kinin pathways [54]. Complement factor H, an essential regulator of the alternative pathway, was also upregulated in chemoresistant samples compared with chemosensitive samples. *In vitro* studies showed decreased levels of this factor in oxidative stress conditions [17]. The interplay between the complement system and oxidative stress has been extensively investigated in age-related macular degeneration [17]. Thurman and colleagues demonstrated that cells exposed to oxidant stress from hydrogen peroxide exhibited decreased levels of complement inhibitors and increased the VEGF expression compared with control cells [55].

Defense mechanisms to avoid MAC (C5b-9 complex) accumulation include the action of vitronectin and clusterin, which were upregulated in the chemoresistant group. Vitronectin and clusterin play critical roles in cell aggregation, complement inhibition, immune signaling regulation, and tissue repair. Together with angiotensin, they may connect oxidative stress to the complement cascade and inflammatory signaling.

Recent findings have suggested that C3, C4, and C5 may aid the survival of tumors through immunosuppression. Other evidence has suggested that complement proteins induce the production of TNF-*α* [56] and TGF-*β* [57, 58] in pathological processes such as cancer. Additionally, complement proteins cooperate with extracellular matrix (ECM) remodeling through the degradation of collagens and gelatins and by activating matrix metalloproteinases (MMPs) [50, 51].

## 5. Conclusion

In summary, the connection among inflammation, the complement system, and oxidative stress described in our study seems to be a pivotal axis in chemoresistance of luminal A breast cancer subtype. These findings will have significant clinical implications for improving BC chemoresistance. Hence, further studies are necessary to determine the main triggers of those signaling pathways in the context of breast cancer. Finally, studies to select molecules that simultaneously inhibit the oxidative and inflammatory pathways are indicated to bypass this chemoresistance in the future.

## Figures and Tables

**Table 1:** Clinicopathological characteristics of the patients.

Variable	
*Total number of patients*	*n* = 200
*Mean age at diagnosis (years)*	56.3
*TNM stage (%)*	
I/II	30%
III/IV	70%
*Tumor histological type (%)*	
Infiltrative ductal carcinoma	100%
*Tumor grade (n*)	
1	5%
2	39%
3	56%
*Tumor size (cm)*	
≤2	15.5%
2–5	54.5%
>5	30%
*Molecular receptor status*	
Positive ER	72%
Positive PR	56%
*Response to chemotherapy*	52%

**Table 2:** Differentially expressed proteins of label-free proteomic analysis.

Upregulated in chemoresistant patients	79
Downregulated in chemoresistant patients	13
Unique in chemoresistant patients	59
Unique in chemosensitive patients	54
Total	205

**Table 3:** Representative biological processes related to proteins upregulated in chemoresistant breast cancer.

*Cytoskeleton remodeling/cell adhesion*
Collagen alpha-1(XII) chain, fibronectin, keratin type I cytoskeletal 10, myosin 7, Rho GTPase-activating protein 35, vitronectin
*Blood coagulation*
Alpha 1-antichymotrypsin, alpha 1-antitrypsin, alpha 2-macroglobulin, antithrombin III, kininogen-1, plasminogen, prothrombin
*DNA repair/kinetochore assembly*
Centromere/kinetochore protein zw10 homolog, DnaJ homolog subfamily C member 10, LINE-1 type transposase domain-containing protein 1
*Oxidative metabolism*
Acetyl-CoA carboxylase 1; activator of 90 kDa heat shock protein ATPase homolog 2; acylpyruvase FAHD1, mitochondrial; afamin; alpha 1B-glycoprotein; alpha 2 HS glycoprotein; angiotensinogen; apolipoprotein E; ceruloplasmin; clusterin; dynein heavy chain 10, axonemal; dynein heavy chain 3, axonemal; exocyst complex component 1; haptoglobin; haptoglobin-related protein; hemoglobin subunit alpha; hemoglobin subunit beta; hemopexin; inositol hexakisphosphate and diphosphoinositol-pentakisphosphate kinase 2; kinectin; nuclear pore complex protein Nup205; nuclear receptor corepressor 2; polypeptide N-acetylgalactosaminyltransferase 3; pregnancy zone protein; prolyl 4 hydroxylase subunit alpha-3; regulator of nonsense transcripts 2; ribose phosphate pyrophosphokinase 3; rod cGMP-specific 3′,5′-cyclic phosphodiesterase subunit alpha; sarcoplasmic/endoplasmic reticulum calcium ATPase 3; serine/threonine protein kinase WNK2; serine/threonine protein phosphatase 2A, 55 kDa regulatory subunit B alpha isoform; serotransferrin; serum albumin; serum amyloid A-4 protein; serum amyloid P component; synembryn-B; transcriptional repressor p66-alpha; vitamin D-binding protein
*Immune response_inflammation*
Alpha-1 acid glycoprotein 1, alpha-1 acid glycoprotein 2, apolipoprotein A-I, apolipoprotein A-II, apolipoprotein B-100, C-reactive protein, inter-alpha-trypsin inhibitor heavy chain H1, inter-alpha-trypsin inhibitor heavy chain H2, inter-alpha-trypsin inhibitor heavy chain H4, lumican, son of sevenless homolog 1, transcription factor 4
*Immune response_humoral immune response*
Ig alpha-1 chain C region, Ig gamma-1 chain C region, Ig gamma-2 chain C region, Ig gamma-3 chain C region, Ig gamma-4 chain C region, Ig heavy chain VI region V35, Ig heavy chain V-II region ARH 77, Ig heavy chain V-III region GAL, Ig heavy chain V-III region TIL, Ig kappa chain C region, Ig kappa chain VI region AU, Ig kappa chain VI region EU, Ig kappa chain VI region Gal, Ig kappa chain VI region Rei, Ig kappa chain V-II region TEW, Ig kappa chain V-III region GOL, Ig kappa chain V-III region NG9 (fragment), Ig kappa chain V-III region SIE, Ig kappa chain V-III region Ti, Ig kappa chain V-III region VG (fragment), Ig lambda-1 chain C regions, Ig lambda-2 chain C regions, Ig lambda-3 chain C regions, Ig mu chain C region, immunoglobulin lambda such as polypeptide 5
*Immune response_complement system*
C4b-binding protein alpha chain, complement C1q subcomponent subunit B, complement C1q subcomponent subunit C, complement C3, complement C4A, Complement C5, complement component C7, complement factor B, complement factor H, plasma protease C1 inhibitor

**Table 4:** Representative biological processes related to downregulated proteins in breast cancer chemoresistance.

*Cytoskeleton remodeling/cell adhesion*
Collagen alpha-1(VII) chain; GRB2-associated-binding protein 1; integrin alpha V; keratin type II cytoskeletal 1; myosin regulatory light chain 2, skeletal muscle isoform; plectin
*Blood coagulation*
Fibrinogen alpha chain, fibrinogen beta chain
*DNA repair/kinetochore assembly*
Centromere protein F, centrosome-associated protein 350, DNA polymerase alpha catalytic subunit, microtubule-associated protein 1B, centrosomal protein of 290 kDa
*Oxidative metabolism*
ATPase family AAA domain-containing protein 3B; dynein heavy chain 1, axonemal; E3 ubiquitin protein ligase UBR5; ectonucleotide pyrophosphatase phosphodiesterase family member 1; glycogen phosphorylase, liver form; inorganic pyrophosphatase; sodium bicarbonate cotransporter 3; TBC1 domain family member 2A; tripeptidyl peptidase 2
*Immune response_inflammation*
Apolipoprotein C-II, RAC-gamma serine/threonine-protein kinase, regulator of G protein signaling 14
*Immune response_humoral immune response*
Ig heavy chain V-III region JON, Ig heavy chain V-III region VH26, Ig kappa chain VI region Roy, Ig lambda chain VI region WAH, Ig lambda chain V-VI region SUT, Ig mu heavy chain disease protein
*Immune response_complement system*
Complement C4B, complement component C8 gamma chain

## Data Availability

The high-throughput proteome data used to support the findings of this study are included within the supplementary information files.

## Supplementary Materials

Supplementary Materials Supplementary Figure 1: interaction networks among the altered proteins in chemoresistant breast cancer identified by Ingenuity Pathway Analysis (IPA) software. Red arrows indicate upregulated proteins and green arrows indicate downregulated proteins in our data. Supplementary Figure 2: histogram from the top canonical pathways among the altered proteins in chemoresistant breast cancer generated using Ingenuity Pathway Analysis (IPA) software. *p* value is indicated on the left side of the histogram. Supplementary Figure 3: in silico analysis obtained with Ingenuity Pathway Analysis (IPA) software showed upregulated proteins in the chemoresistant breast cancer group identified in the complement system cascade pathway. Pink-shaded proteins were upregulated in chemoresistant patients. Supplementary Figure 4: Supplementary Figure Y: coagulation system pathway identified with Ingenuity Pathway Analysis (IPA) software. Shaded notes represented altered proteins presented in chemoresistant breast cancer. Pink-shaded objects indicate upregulated proteins, and green-shaded objects indicate downregulated proteins. Supplementary Figure 5: production of nitric oxide and reactive oxygen species signaling pathway identified using Ingenuity Pathway Analysis (IPA) software. Pink-shaded target indicates upregulated proteins and green-shaded target indicates downregulated proteins presented in chemoresistant breast cancer. Supplementary Figure 6: actin cytoskeleton signaling pathway identified using Ingenuity Pathway Analysis (IPA) software. Pink-shaded target indicates upregulated proteins and green-shaded target indicates downregulated proteins presented in chemoresistant breast cancer.
