# Supplementary material for: Label-Free Proteomics Revealed Oxidative Stress and Inflammation as Factors That Enhance Chemoresistance in Luminal Breast Cancer
Source: Oxid Med Cell Longev. 2019 Aug 8;2019:5357649. doi: 10.1155/2019/5357649 (PMC6702830; doi:10.1155/2019/5357649)
Supplement: Supplementary Materials — Supplementary Figure 1: interaction networks among the altered proteins in chemoresistant breast cancer identified by Ingenuity Pathway Analysis (IPA) software. Red arrows indicate upregulated proteins and green arrows indicate downregulated proteins in our data. Supplementary Figure 2: histogram from the top canonical pathways among the altered proteins in chemoresistant breast cancer generated using Ingenuity Pathway Analysis (IPA) software. p value is indicated on the left side of the histogram. Supplementary Figure 3: in silico analysis obtained with Ingenuity Pathway Analysis (IPA) software showed upregulated proteins in the chemoresistant breast cancer group identified in the complement system cascade pathway. Pink-shaded proteins were upregulated in chemoresistant patients. Supplementary Figure 4: Supplementary Figure Y: coagulation system pathway identified with Ingenuity Pathway Analysis (IPA) software. Shaded notes represented altered proteins presented in chemoresistant breast cancer. Pink-shaded objects indicate upregulated proteins, and green-shaded objects indicate downregulated proteins. Supplementary Figure 5: production of nitric oxide and reactive oxygen species signaling pathway identified using Ingenuity Pathway Analysis (IPA) software. Pink-shaded target indicates upregulated proteins and green-shaded target indicates downregulated proteins presented in chemoresistant breast cancer. Supplementary Figure 6: actin cytoskeleton signaling pathway identified using Ingenuity Pathway Analysis (IPA) software. Pink-shaded target indicates upregulated proteins and green-shaded target indicates downregulated proteins presented in chemoresistant breast cancer. [file 5357649.f1.zip › Supplementary_files_R1.doc]

# SUPPLEMENTARY MATERIAL

**Graphical Abstract**

In brief: Label-free mass spectrometry-based proteomics as a strategy for studying the differences between patients with responsive and chemoresistant breast cancer. The results highlight inflammatory and oxidative stress signaling in breast cancer chemoresistance.

**Supplementary Figure 1.** Interactions networks among the altered proteins in chemoresistant breast cancer identified by Ingenuity Pathway Analysis (IPA) software. Red arrows indicate upregulated proteins and green arrows indicate downregulated proteins in our data.

**Supplementary Figure 2.** Histogram from the top canonical pathways among the altered proteins in chemoresistant breast cancer generated using Ingenuity Pathway Analysis (IPA) software. P-value are indicated on the left side of the histogram.

**Supplementary Figure 3.** *In silico* analysis obtained with Ingenuity Pathway Analysis (IPA) software showed upregulated proteins in chemoresistant breast cancer group identified in Complement system cascade pathway. Pink shaded proteins were upregulated in chemoresistant patients.

**Supplementary Figure 4.** Supplementary Figure Y. Coagulation system pathway identified with Ingenuity Pathway Analysis (IPA) software. Shaded notes represented alterated proteins presented in chemoresistant breast cancer. Pink shaded objets indicate upregulated proteins and green shaded objects indicate downregulated proteins.

**Supplementary Figure 5.** Production of Nitric Oxide and Reactive Oxigen Species signaling pathway identified using Ingenuity Pathway Analysis (IPA) software. Pink shaded target indicates upregulated proteins and green shaded target indicates downregulated proteins presented in chemoresistant breast cancer.

**Supplementary Figure 6.** Actin Cytoskeleton signaling pathway identified using Ingenuity Pathway Analysis (IPA) software. Pink shaded target indicates upregulated proteins and green shaded target indicates downregulated proteins presented in chemoresistant breast cancer.
